# Dynamic association rules for gene expression data analysis

**DOI:** 10.1186/s12864-015-1970-x

**Published:** 2015-10-14

**Authors:** Shu-Chuan Chen, Tsung-Hsien Tsai, Cheng-Han Chung, Wen-Hsiung Li

**Affiliations:** Department of Mathematics and Statistics, Idaho State University, Pocatello, ID 83209 USA; Department of Statistics, National Cheng-Kung University, Tainan, 701 Taiwan; Department of Biological Sciences, Idaho State University, Pocatello, ID 83209 USA; Academia Sinica, Taipei, 115 Taiwan; Department of Ecology and Evolution, University of Chicago, Chicago, IL 60637 USA

**Keywords:** Association rules, Gene expression data, Bioinformatics, Data mining, Transcriptome analysis

## Abstract

**Background:**

The purpose of gene expression analysis is to look for the association between regulation of gene expression levels and phenotypic variations. This association based on gene expression profile has been used to determine whether the induction/repression of genes correspond to phenotypic variations including cell regulations, clinical diagnoses and drug development. Statistical analyses on microarray data have been developed to resolve gene selection issue. However, these methods do not inform us of causality between genes and phenotypes. In this paper, we propose the dynamic association rule algorithm (DAR algorithm) which helps ones to efficiently select a subset of significant genes for subsequent analysis. The DAR algorithm is based on association rules from market basket analysis in marketing. We first propose a statistical way, based on constructing a one-sided confidence interval and hypothesis testing, to determine if an association rule is meaningful. Based on the proposed statistical method, we then developed the DAR algorithm for gene expression data analysis. The method was applied to analyze four microarray datasets and one Next Generation Sequencing (NGS) dataset: the Mice Apo A1 dataset, the whole genome expression dataset of mouse embryonic stem cells, expression profiling of the bone marrow of Leukemia patients, Microarray Quality Control (MAQC) data set and the RNA-seq dataset of a mouse genomic imprinting study. A comparison of the proposed method with the *t*-test on the expression profiling of the bone marrow of Leukemia patients was conducted.

**Results:**

We developed a statistical way, based on the concept of confidence interval, to determine the minimum support and minimum confidence for mining association relationships among items. With the minimum support and minimum confidence, one can find significant rules in one single step. The DAR algorithm was then developed for gene expression data analysis. Four gene expression datasets showed that the proposed DAR algorithm not only was able to identify a set of differentially expressed genes that largely agreed with that of other methods, but also provided an efficient and accurate way to find influential genes of a disease.

**Conclusions:**

In the paper, the well-established association rule mining technique from marketing has been successfully modified to determine the minimum support and minimum confidence based on the concept of confidence interval and hypothesis testing. It can be applied to gene expression data to mine significant association rules between gene regulation and phenotype. The proposed DAR algorithm provides an efficient way to find influential genes that underlie the phenotypic variance.

## Background

Applications of the association rule have been made across multidisciplinary fields [1, 2]. In biological sciences, it has been applied to analyze gene expression data. For example, Becquet et al. [3] developed the Min-Ex algorithm which applied on human SAGE data. This algorithm can be used for mining rules in dense boolean matrices to eliminate redundant association rules. Since explore association rules is a huge computational work when boolean matrices is large, their algorithm also efficiently reduces the size of the search space. Creighton and Hanash [4] implemented a database application with the Apriori algorithm [5] which is often used to explore association rules. They applied this algorithm to mine association between genes. These two examples could identify rules that contained a set of proteins with potential interactions. Park et al. [6] applied fuzzy association rule mining techniques to handle continuous numerical values in time series microarray data. It successfully discovered several gene expression patterns over times that were supported by yeast cell cycle data.

The goal of association rules mining is to establish the relationship between a set of input variables and a set of output variables. Some packages have been developed for computational purpose. For example, Hahsler et al. [7] developed an R package, called ‘arules’, to manipulate input data sets and analyze the resultant item sets and rules. Palanisamy [8] modified a well-known association rule mining algorithm, Apriori, to deal with input constraints. Two important indices for an association rule are *support* and *confidence*. For any association rule to be meaningful, it is critical to have sufficiently high values of support and confidence.

In data mining, currently the thresholds for the support and the confidence are set arbitrarily by users, so that different sets of support and confidence will lead to different results. Users have to try different settings in order to have a better result. Therefore, the results are difficult to interpret. How to determine the thresholds remains an important issue in association rules mining.

In this paper, we propose a statistical way based on the concept of confidence interval and hypothesis testing to determine the minimum support and the minimum confidence for an association rule. We also show how to estimate the minimal support and the minimal confidence for a given set of data. A rule is meaningful only if the support and the confidence are both significantly greater than the minimal thresholds. We then apply the method to gene expression data analysis.

Gene expression analysis is a common way to characterize gene expression profiles and to identify a specific expression profile(s) associated with a disease or trait. DNA microarrays were commonly used in gene expression analysis before the Next Generation Sequencing era. A DNA microarray is a collection of DNA probes which are orderly attached on a solid surface [9]. Each probe represents a detector for a gene with a distinct sequence. Therefore, a whole-genome expression microarray can measure the expression level of every gene on the chip at once [10]. The Next Generation Sequencing (NGS) technology has advanced rapidly since the first RNA-seq paper was published in 2006 [11] and has almost completely replaced the microarray approach. Instead of studying expression profiles by hybridizing with known probes on microarrays, one can acquire the actual RNA sequences expressed in cells using a NGS platform [12, 13]. For RNA-seq data, the expression level of a gene can be expressed in terms of Reads Per Kilobase per Million mapped reads (RPKM) [12] or Fragments Per Kilobase per Million mapped fragments (FPKM) [14]. To take account of differences in the coverage/depth of reads in different samples or experiments, normalization and bias correction methods have been developed [15]. The differential expression of a gene between experimental conditions could then be assessed using statistical tests, e.g., the t-statistics or the likelihood ratio test based on a generalized linear model.

In gene expression analysis, a popular issue is to look for a set of significant genes corresponding to a specific phenotype. Researchers use gene expression data to determine whether the induction/repression of a gene is informative to diagnose the symptoms of a disease [16–18]. Statistics play an important role in such a gene selection problem. The main statistical techniques currently used are multiple t-tests, factor analysis, principle component analysis and other dimension reduction methods [19]. These methods can extract a subset of genes that are relevant to the disease or phenotype. However, they do not inform us of causality between genes and phenotypes. Therefore, we propose the *dynamic association rule algorithm *(described in Fig. 3), called DAR algorithm, which preserves the causality of genes and symptoms.

**Fig. 1:**
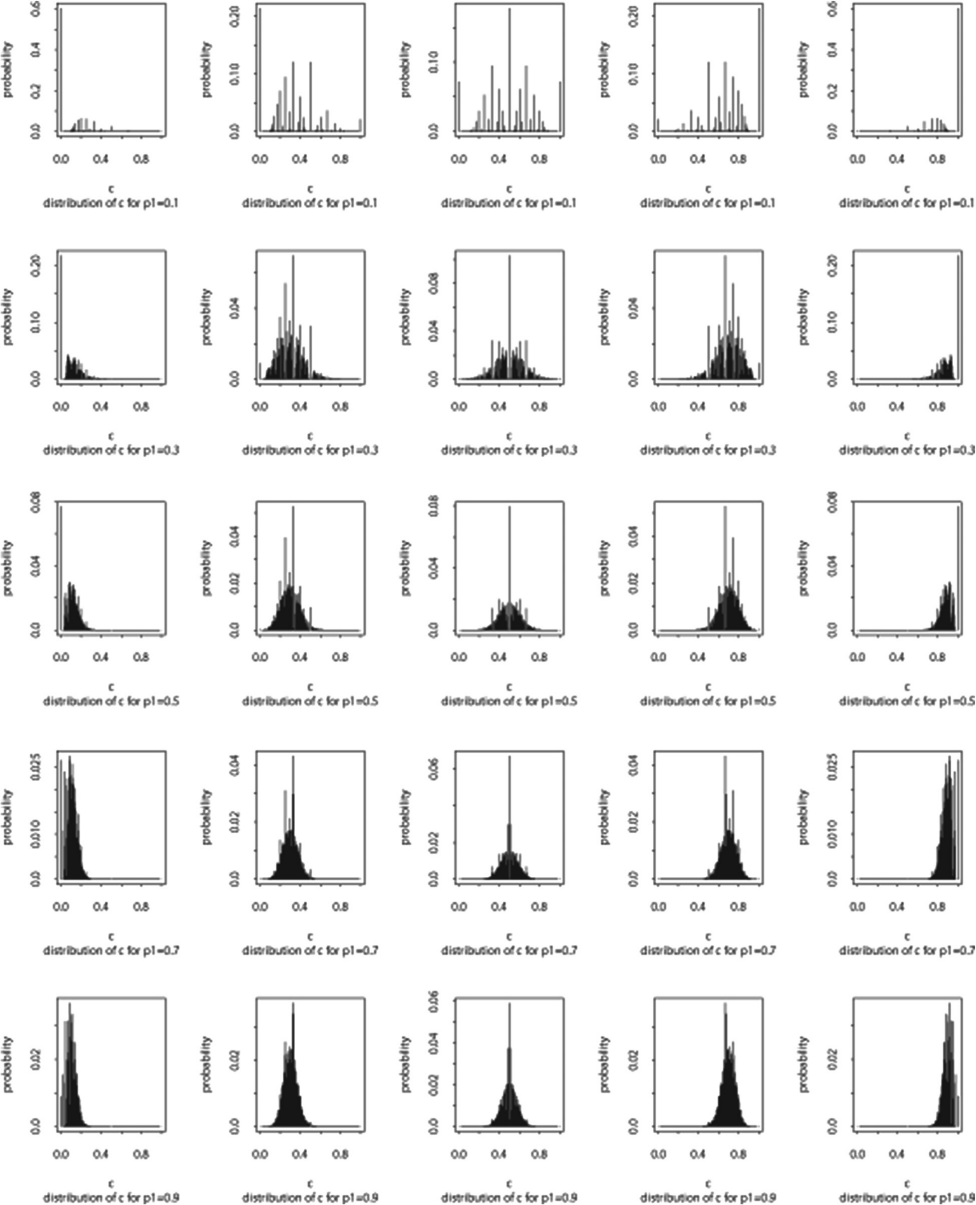
The distribution of confidence for all combinations of p_1_ = (0.1, 0.3, 0.5, 0.7, 0.9) and p_2_ = (0.1, 0.3, 0.5, 0.7, 0.9) under independence when *n* = 50

First, we establish all possible association rules between gene expression levels and phenotypes. For example, one of the specific association rules can be expressed as follows:

$$ +1\mathrm{g}\mathrm{e}\mathrm{n}\mathrm{e}\mathrm{A}\to \mathrm{dis}\mathrm{e}\mathrm{a}\mathrm{s}\mathrm{e}\ \mathrm{g}\mathrm{roup}. $$

This rule indicates that if gene A is over-expressed, labeled as +1geneA, then it implies a disease group. Here “imply” means “causation”. It gives us information in biological sense. Support and confidence are used to measure the strength of an association rule. Here support measures the joint probability of +1geneA and the phenotype, while confidence measures the conditional probability of observing the phenotype given +1geneA.

Second, we apply the minimum support and minimum confidence concept to find “significant rules”.

Third, we screen out those impossible (ambiguous) rules. For example, if both rules, “-1geneA, 1gene B → disease group” and “-1geneA, 1gene B → non disease group”, are significant, we consider them *ambiguous rules* because the two rules are conflicting.

Finally, we construct the final rules and determine the final significant genes

With the proposed DAR algorithm, we can find a set of genes that are related to the phenotypic difference between groups. We demonstrate the approach using microarray datasets for High-density lipoprotein (HDL)-Deficient mice [19], mouse embryonic stem cells (ESC) [20], and the bone marrow of Leukemia patients [21], and the RNA-seq data from a mouse genomic imprinting study [22]. In all four applications, our proposed method can capture the influential genes and the underlying biological functions known in literature, and can provide additional influential genes that other methods failed to identify. In addition, a comparison with *t*-test is made, using the expression data from the bone marrow of Leukemia patients [21].

## Methods

### Model

Let I = (i_1_, …, i_k_) be a set of k elements, called items. A basket dataset B = (b_1_, …, b_n_) is any collection of n subsets of I and each subset b_i_ ⊆ I is called a basket of items. Given support s and confidence c, we say there is an established association rule [2]

A (called antecedent) → B (called consequent) if

a. A and B occur together in at least s · 100 % of the n baskets.

b. Among those baskets containing A, at least c · 100 % also contain B.

Here

$$ \mathrm{support}=\frac{\mathrm{transactions}\ \mathrm{that}\ \mathrm{contain}\ \mathrm{every}\ \mathrm{item}\ \mathrm{in}\ \mathrm{A}\ \mathrm{and}\ \mathrm{B}}{\mathrm{all}\ \mathrm{transactions}} $$

and

$$ \mathrm{confidence}=\frac{\mathrm{transactions}\ \mathrm{that}\ \mathrm{contain}\ \mathrm{every}\ \mathrm{item}\ \mathrm{in}\ \mathrm{A}\ \mathrm{and}\ \mathrm{B}}{\mathrm{transactions}\ \mathrm{that}\ \mathrm{contain}\ \mathrm{the}\ \mathrm{item}\mathrm{s}\ \mathrm{in}\ \mathrm{A}}. $$

Mathematically stated, the confidence is the probability that the items in the antecedent A appear together with the items in the consequent B. The significance of an association relationship between A and B can be measured by the support and the confidence. The probability representations of the support and the confidence are,

$$ \mathrm{support}=\mathrm{P}\left(\mathrm{A}{\displaystyle \cap}\mathrm{B}\right),\ \mathrm{and}\ \mathrm{confidence}=\mathrm{P}\left(\mathrm{B}\Big|\mathrm{A}\right) $$

Consider the association rule A → B. Let n be the total number of items in the population, n_A_ be the total number of items in A, and n_AB_ be the total number of items in both A and B. Then numerically the support and the conference are estimated as follows:

$$ \mathrm{support}=\frac{{\mathrm{n}}_{\mathrm{A}\mathrm{B}}}{\mathrm{n}},\ \mathrm{and}\ \mathrm{confidence}=\frac{{\mathrm{n}}_{\mathrm{A}\mathrm{B}}}{{\mathrm{n}}_{\mathrm{A}}} $$

Currently, the thresholds of the support and the confidence are set arbitrarily by users and it is very difficult to interpret the result. If the thresholds of the support and the confidence are set too low, many rules will be established. On the other hand, if the thresholds are set too high, no rules may be established. Therefore, how to determine the thresholds of the support and the confidence becomes an important issue in the study of the association rules mining. The idea behind our proposed algorithm is as follows. When the antecedent follows a specific distribution, we can first compute the distributions of the support and the confidence, and then determine the thresholds of the support and the confidence by the concept of confidence interval. All rules have to meet the minimal support and the minimal confidence in order to be meaningful.

Suppose indicator functions I_A_ ~ Bernoulli (p_1_) and I_B_ ~ Bernoulli (p_2_). Under the independence assumption, we have the indicator function I_AB_ ~ Bernoulli (p_1· _p_2__._). Let n, n_A_, n_B_ and n_AB_ be the total number of items in the population, the total number of items in A, the total number of items in B, and the total number of items in both A and B, respectively. We can derive the distribution of the support (s) as follows.

$$ \mathrm{s}=\frac{{\mathrm{n}}_{\mathrm{AB}}}{\mathrm{n}}\sim \frac{1}{\mathrm{n}}\mathrm{Bin}\left(\mathrm{n},{\mathrm{p}}_{12}\right)\sim \frac{1}{\mathrm{n}}\mathrm{N}\left({\mathrm{n}\mathrm{p}}_{12},\sqrt{{\mathrm{n}\mathrm{p}}_{12}\left(1-{\mathrm{p}}_{12}\right)}\right)\sim \mathrm{N}\left({\mathrm{p}}_{12},\sqrt{\frac{{\mathrm{p}}_{12}\left(1-{\mathrm{p}}_{12}\right)}{\mathrm{n}}}\right). $$

Here Bin(n, p_12_) stands for the Binomial distribution with parameters n and p_12_, and $\mathrm{N}\left({\mathrm{np}}_{12},\ \sqrt{{\mathrm{np}}_{12}\left(1-{\mathrm{p}}_{12}\right)}\right)$ stands for the Normal distribution with mean np_12_ and standard deviation $\sqrt{{\mathrm{np}}_{12}\left(1-{\mathrm{p}}_{12}\right)}$. Note that, such an approximation is in general appropriate because we are dealing with a large n (even though p may be small). See, for example, Arnold (1990, p. 143) [23]. Therefore, the theoretical minimal support under the assumption of independence (p_12_ = p_1_ · p_2_) is its (1 − α) · 100 % upper bound, with α being the statistical significant level.

That is,

1 $$ {\mathrm{p}}_1{\mathrm{p}}_2+{\mathrm{z}}_{\mathrm{a}}\sqrt{\frac{{\mathrm{p}}_1{\mathrm{p}}_2\left(1-{\mathrm{p}}_1{\mathrm{p}}_2\right)}{\mathrm{n}}} $$

For the distribution of confidence, suppose X is the number of A ∩ B, then X ∼ Bin (n, p_12_), which is the Binomial distribution with parameters n and p_12_. Let Z be the number of A, then Z ∼ Bin (n, p_1_). The confidence, c, then becomes C = X/Z. Technically, C is a defective random variable, as the event Z = 0 is ignored here. Because the probability P (Z = 0) is rather small, the truncated distribution is nearly identical to the un-truncated distribution. It is known that the distribution of X given Z = z is a Binomial distribution with parameters z and p_2|1_, where p_2|1_ is the conditional probability of B given A.

Under the independence assumption, for any specific value c, we have

$$ \mathrm{P}\left(\frac{\mathrm{X}}{\mathrm{Z}}=\mathrm{C}=\mathrm{c}\right)={\displaystyle \sum_{\mathrm{z}}}\mathrm{P}\left(\frac{\mathrm{X}}{\mathrm{Z}}=\mathrm{c}\Big|\mathrm{Z}=\mathrm{z}\right)\mathrm{P}\left(\mathrm{Z}=\mathrm{z}\right)={\displaystyle \sum_{\mathrm{z}}}\mathrm{P}\left(\mathrm{x}=\mathrm{c}\mathrm{z}\Big|\mathrm{Z}=\mathrm{z}\right)\mathrm{P}\left(\mathrm{Z}=\mathrm{z}\right)={\displaystyle \sum_{\mathrm{z}=1}^{\mathrm{n}}}\mathrm{Bin}\left(\mathrm{z},\mathrm{c}\mathrm{z},{\mathrm{p}}_2\right)\mathrm{Bin}\left(\mathrm{n},\mathrm{z},{\mathrm{p}}_1\right) $$

where Bin (z, cz, p_2_) = $$ \left(\begin{array}{c}\hfill \mathrm{z}\hfill \\ {}\hfill \mathrm{c}\mathrm{z}\hfill \end{array}\right){\mathrm{p}}_2^{\mathrm{cz}}{\left(1-{\mathrm{p}}_2\right)}^{\mathrm{z}-\mathrm{c}\mathrm{z}} $$ and Bin (n, z, p_1_) = $$ \left(\begin{array}{c}\hfill \mathrm{n}\hfill \\ {}\hfill \mathrm{z}\hfill \end{array}\right){\mathrm{p}}_1^{\mathrm{z}}{\left(1-{\mathrm{p}}_1\right)}^{\mathrm{n}-\mathrm{z}} $$.

Unlike the distribution for the support, the minimum confidence does not have a direct formula like (1); however, numerical results are always possible and will be illustrated below.

**Fig. 2:**
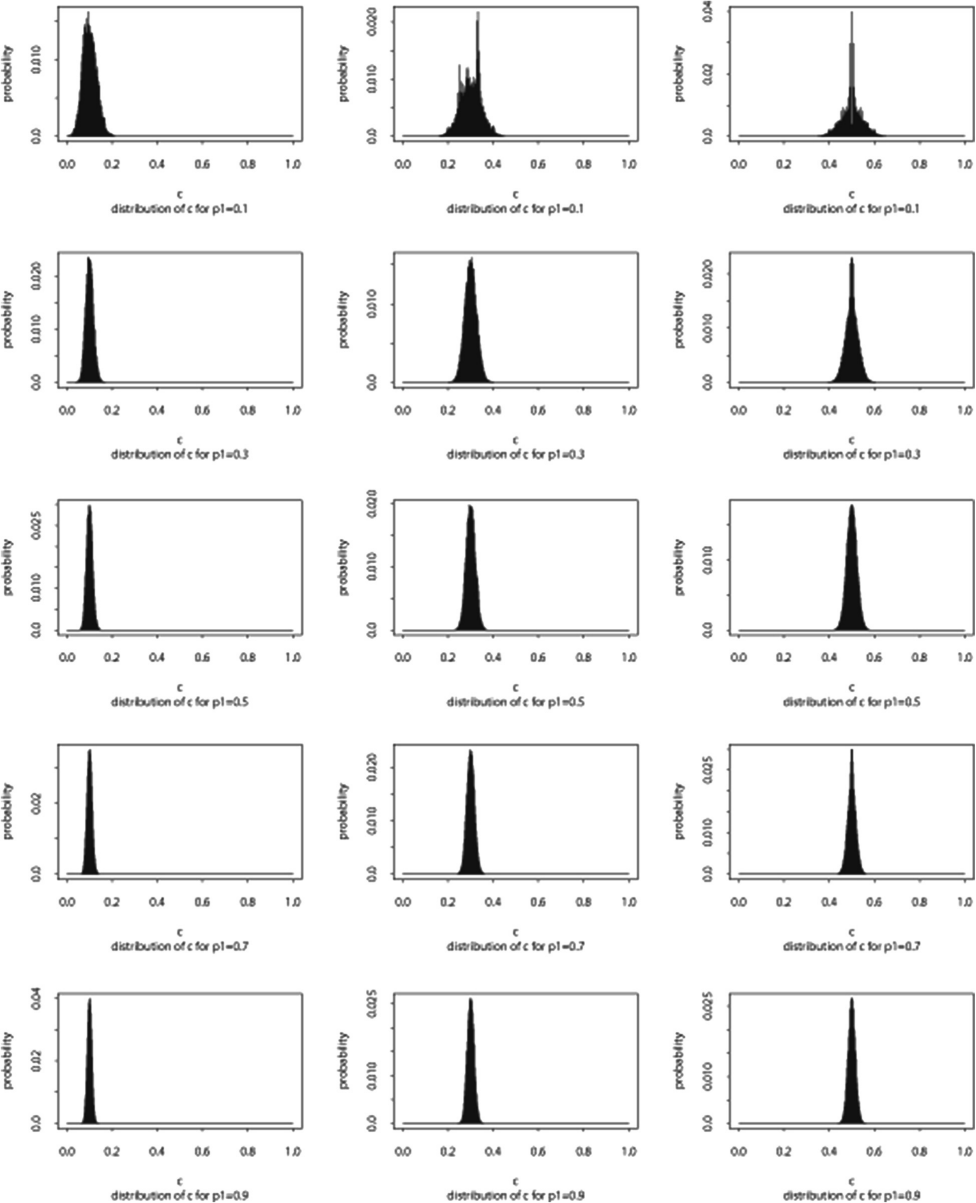
The distribution of confidence under independence when *n* = 1000

For example, the distribution of the confidence is the sum of product of two binomials. Figure 1 displays the exact distribution for all combinations of p_1_ = (0.1, 0.3, 0.5, 0.7, 0.9) and p_2_ = (0.1, 0.3, 0.5, 0.7, 0.9) when *n* = 50. It is clear that the distribution is symmetric in p_1_. That is, given a fixed p_1=_p_0_, the distribution of the confidence when p_2_ = p^*^ is the same with the distribution of (1-confidence) when p_2_ = (1-p^*^). For example, as in Fig. 1, given p_1_ = 0.1 (first row), the distribution of confidence for p_2_ = 0.1 is identical to the distribution of confidence for p_2_ = 0.9. Likewise, the distribution of confidence for p_2_ = 0.3 is symmetric of confidence for p_2_ = 0.7. Furthermore, for larger n, the distribution is approximately normal as can be seen in Fig. 2.

The variance of confidence (X/Z) is p_2_(1-p_2_)E(1/Z). When n is large enough, $Z\sim N\left(n{p}_1,\ \sqrt{n{p}_1\left(1-{p}_1\right)}\right).$ It can be shown that E(1/Z) does not exist, and thus Var (X/Z) does not exist. Therefore, we can only resolve the problem numerically. For example, the minimum confidences are tabulated in Table 1 for *n* = 1000. For large n (>1000), the values given in Table 1 can be a good approximation.

**Table 1:** Critical value when *n* = 1000

				p_1_			
			0.1	0.3	0.5	0.7	0.9
	0.1	80 %	0.1245	0.1135	0.1105	0.1095	0.1075
		90 %	0.1395	0.1225	0.1165	0.1145	0.1125
		95 %	0.1515	0.1285	0.1225	0.1185	0.1165
		99 %	0.1755	0.1415	0.1315	0.1265	0.1235
	0.3	80 %	0.3385	0.3215	0.3165	0.3145	0.3125
		90 %	0.3585	0.3335	0.3255	0.3215	0.3195
		95 %	0.3755	0.3435	0.3335	0.3285	0.3245
		99 %	0.4095	0.3625	0.3475	0.3405	0.3355
p_2_	0.5	80 %	0.5415	0.5235	0.5185	0.5155	0.5135
		90 %	0.5635	0.5365	0.5285	0.5235	0.5205
		95 %	0.5825	0.5475	0.5365	0.5305	0.5265
		99 %	0.6165	0.5665	0.5515	0.5435	0.5385
	0.7	80 %	0.7385	0.7215	0.7165	0.7145	0.7125
		90 %	0.7585	0.7335	0.7255	0.7215	0.7195
		95 %	0.7745	0.7425	0.7325	0.7275	0.7245
		99 %	0.8035	0.7605	0.7465	0.7395	0.7345
	0.9	80 %	0.9255	0.9145	0.9105	0.9095	0.9075
		90 %	0.9375	0.9215	0.9165	0.9135	0.9125
		95 %	0.9465	0.9275	0.9215	0.9175	0.9155
		99 %	0.9635	0.9375	0.9295	0.9255	0.9225

### Example

Given a dataset of size *n*, to verify any association rule of “if A then B”, in general, we first obtain the following estimates

$$ {\widehat{p}}_A=\frac{n_A}{n} $$

and

$$ {\widehat{p}}_B=\frac{n_B}{n} $$

where *n*_*A*_ is the number of records that belongs to the Event A. Likewise, *n*_*B*_ is the number of records that containing Event B. The minimum support is then

$$ {s}_{min}={\widehat{p}}_A{\widehat{p}}_B+{z}_{\alpha}\sqrt{\frac{{\widehat{p}}_A{\widehat{p}}_B\left(1-{\widehat{p}}_A{\widehat{p}}_B\right)}{n}} $$

and the minimal confidence can be obtained from Table 1.

For example, a dataset of size *n* = 1000 with *n*_*A*_ = 300 and *n*_*B*_ = 700. We will have

$$ {\widehat{p}}_A=\frac{300}{1000}=0.3 $$ and $$ {\widehat{p}}_B=\frac{700}{1000}=0.7 $$.

Thus,

$$ {s}_{min}=0.3\times 0.7+1.645\sqrt{\frac{0.3\times 0.7\left(1-0.3\times 0.7\right)}{1000}}=0.2312 $$

and from Table 1, the *minimum confidence* = 0.7425.

Any association rule must beat these minimal support and minimal confidence values to be meaningful.

## Results and discussion

**Fig. 3:**
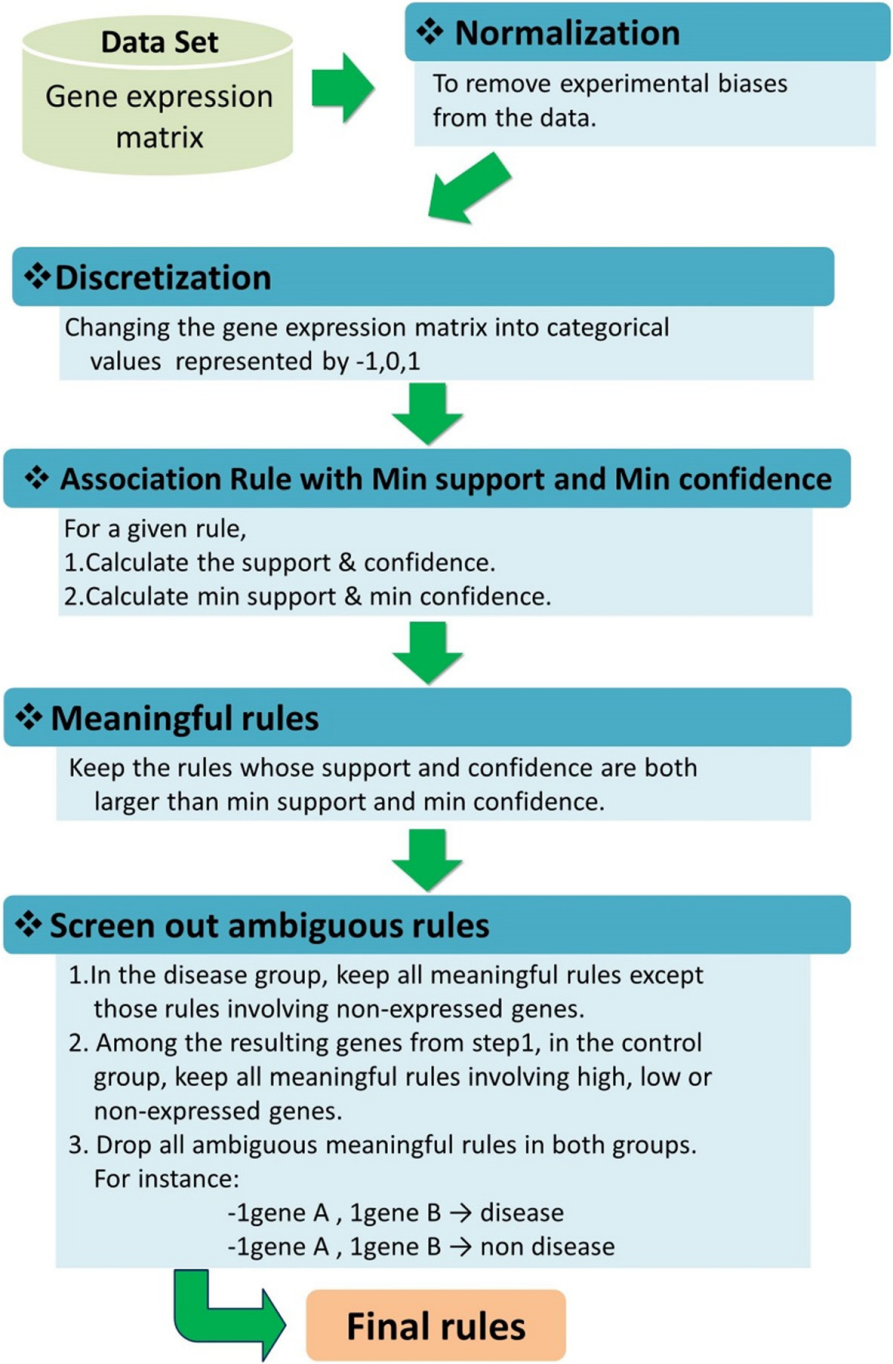
The DAR algorithm for microarray data analysis

First, we normalized gene expression data to remove systematic bias. Next, we re-labeled expression levels with categorical variables. For example, in the ApoA1 knockout mice data [19], we applied lowess fit on the expression data for normalization [24]. Then we re-labeled the expression level with −1 (low expression), 0 (no expression), and 1 (high expression). For a specific rule, we first calculated the estimated support and confidence from the data, and the theoretical minimum support and the theoretical minimum confidence from the methods that are described in the Method section. We kept those rules whose estimated support and confidence are, respectively, larger than the theoretical minimum support and the theoretical minimum confidence. We then removed those ambiguous genes and kept the remaining genes as the final set of genes for further analysis. The steps of the algorithm proposed in the paper are shown in Fig. 3.

### Analysis of the ApoA1-knockout mice data [19]

The dataset was collected from the ApoA1 knockout experiment of Callow et al. [19]. Apolipoprotein A1 (ApoA1) is a gene known to play a pivotal role in HDL. Callow et al. [19] identified the underlying genes and pathways using ApoA1 knockout mice. The purpose was to identify genes with altered expression in the livers of ApoA1 knockout mice compared to the control inbred mice. A total of 16 mice were divided into a group of 8 control mice and a group of 8 ApoA1 knockout mice. There were 6384 genes involved in this study. To conduct the association rules analysis, we preprocess the data. The first step is normalization and we follow the method proposed by Callow et al. [19]:

$$ { \log}_2\raisebox{1ex}{$R$}\!\left/ \!\raisebox{-1ex}{$G$}\right.\to { \log}_2\raisebox{1ex}{$R$}\!\left/ \!\raisebox{-1ex}{$G$}\right.-{c}_j(A), $$

where A = ${ \log}_2\sqrt{\mathrm{RG}}$ and cj (A) is the lowess fit for log_2_ R/G vs. A.

The second step is to discretize the data. If the normalized log value is ≧1, we label it as 1; if it is ≦ − 1, we label it as −1; and if it is between −1 and 1, we label it as 0. If the expression level is labeled as 1, we classify the gene as “over expressed” in this sample; if the expression level is labeled as −1, we classify the gene as “under expressed”; all other genes are “not differentially expressed”.

Given a threshold setting of the confidence and the support (the default setting is support s = 0.1 and confidence c = 0.85), the well-known Apriori software automatically tries all possible association rules and keeps those rules with the support and the confidence higher than the respective thresholds. With different combinations of the threshold settings of the confidence (from 80 % to 85 %) and the support (from 10 % to 30 %), we wrote the Matlab codes to run the single antecedent analysis and the double antecedent analysis. The numbers of resulting rules are listed in Table 2. Table 2 shows that the number of meaningful rules decreases rapidly as the support increases from 10 % to 20 %.

**Table 2:** The results of the single antecedent rules mining when the DAR algorithm is applied to the data of Callow et al. [19]

Support/Confidence	Meaningful rules	Screen rules	Final genes
10/80	216	198	198
10/85	216	198	198
10/90	215	197	197
20/80	20	14	14
20/85	20	14	14
20/90	19	13	13
30/80	14	9	9
30/85	14	9	9
30/90	13	8	8
Dynamic Support and Confidence	14	9	9

**Table 3:** The results of the double antecedent rules mining when the DAR algorithm was applied to the data of Callow et al. [19]

Support/ Confidence	Meaningful rules	Screen rules	Final genes
10/80	1,298,658	23,076	454
10/85	1,298,224	23,075	454
10/90	1,298,010	23,074	454
20/80	120,241	312	52
20/85	119,807	311	52
20/90	113,393	310	52
30/80	83,228	65	14
30/85	82,794	64	14
30/90	76,830	63	14
Dynamic Support and Confidence	82,361	64	14

Next, we applied our method to compute all possible rules that passed the estimated theoretical minimal support and minimal confidence. Some rules are not meaningful in biology, so we classified them as ambiguous and removed them. For example, if one rule tells us “highly expressed gene A implies disease significantly”, but another rule tells us “highly expressed gene A implies control significantly”, then they are conflicting and ambiguous for biological interpretation. We screened out such ambiguous rules.

Figure 3 shows the flowchart from normalizing the microarray data, discretizing the raw data, applying the DAR method to obtain the meaningful rules, and then screening out the ambiguous rules. The results of applying our method to the DHL-deficient mice data are given in Table 2. It shows that the number of meaningful rules changes due to the different combinations of the cutoff points of support and confidence. With the dynamic support and confidence, however, it gives the number of meaningful rules to be 14, which can be found when the cutoff points were set to be *s* = 30/*c* = 85. The number of the final significant genes is 9.

The result of single antecedent rules mining using the DAR algorithm is listed in Table 2. It shows that the gene expression level of HDL-deficient mice had significant differences in 14 Expressed Sequence Tags (ESTs). Five of them violated the ambiguous rules and were eliminated from the final result. In the published mice data analysis, 8 ESTs were identified to be influential in HDL-deficient mice [24]. Importantly, all 8 ESTs that were found to be significantly influential in the source study [24] were obtained in our study (Table 4). There were four underlying genes for the influential ESTs, which were ApoA1, ApoCIII, Sterol C5 desaturase (SC5D) and spermatogenesis associated 5-like 1 (SPATA5L1). The most underrepresented gene that was kept under the dynamic rule association was the ApoA1 gene. The expression level of ApoA1 in the knockout mice was 20-fold lower than that in the control mice [19]. The expression level of the ApoCIII gene, only 4 kb away from the ApoA1 gene on chromosome 9, was also decreased. SC5D is a catalase that dehydrogenizes C5-6 of lathosterol into double bonds in cholesterol synthesis [25]. EST ID 1496, known as spermatogenesis associated 5-like 1 (Spata5l1), is a member of the ATPase protein family. In addition, a new EST associated with ApoA1 knockout, caspase 6 (CASP6), was significant in a single antecedent with an under-represented expression level in knockout mice. CASP6 is one of the caspase families that play a central role of proteolytic activities during cell apoptosis. It has been found that the oxidized LDLs (oxLDLs) can trigger ER stress and lead to dysfunction and apoptosis of cells [26]. On the other hand, HDLs display protective effects against oxLDLs toxicity. This may imply that cells are easier to undergo apoptosis in the HDL deficient model. However, the regulation of effector caspase CASP6 and the role in HDL deficiency need to be clarified by more experiments. Here the DAR algorithm shows high reproducibility of the original experimental data.

**Table 4:** The influential genes found when the DAR algorithm was applied to the data in Callow et al. [19]

Rule type	ID	Gene	Expression level	Influential in S. Dudoit 2002	Multiple *t*-test (adj. *p*-value)
Single Antecedent	540	Apo AI	−1	Yes	4.00E−04
	1496	SPATA5L1	−1	Yes	0.0156
	1739	Apo CIII	−1	Yes	4.00E−04
	2149	Apo AI	−1	Yes	4.00E−04
	2537	Apo CIII	−1	Yes	7.00E−04
	4139	SC5D	−1	Yes	5.00E−04
	4941	SC5D	−1	Yes	0.0086
	5356	Apo AI	−1	Yes	7.00E−04
	2296	CASP6	−1	New	0.4745
Additional in Double antecedent	5053	BLANK	1	New	1
	5419	DTNBP1	1	New	1
	6215	MAK	1	New	1
	6245	BLANK	1	New	1
	6379	CAS1	1	New	1

The results of the double antecedent association rules mining can be found in Table 3. It shows that 82,361 rules had passed the threshold of the DAR algorithm. After screening out those ambiguous rules, only 14 final rules remained and they included all 9 influential ESTs in the result of the single antecedent rules mining. The result of the double antecedent rules mining enhances the significance of the dynamic association rules mining in biological meaning. Two of the five additional ESTs were “BLANK” in the hybridized microarrays, implying no expression sequence (Table 4). There were 840 BLANK cells in each array. These cells would also be detected and transferred into intensities as background signals. In our normalization, there is still a chance to be significantly different in very low intensity but in a large ratio between knockouts and controls stochastically. One interesting additional EST is EST ID 6379, which represents catalase (CAS1) and plays a major role as antioxidant enzyme against oxidative stress. Catalase converts the hydrogen peroxide to water and oxygen for alleviating the toxicity in cells. It was noticed that HDL prevented the increase of intracellular reactive oxygen through catalase activity, decreasing EGFR activation triggered by oxidized low-density lipoprotein (oxLDL) and H_2_O_2_ [27]. It implies that catalase is regulated by HDL. When the HDL level decreases, the expression of catalase should be up-regulated to compensate the antioxidant activity and also lose the control by HDL regulation. We also apply multiple t test with adjusted p-value. The results are listed in Table 4. In Table 4, dystrobrevin binding protein 1 (DTNBP1), known as dysbindin, is a protein of dystrophin-associated protein complex (DPC) in skeletal muscle cells. It is also a part of lysosome-related organelles complex 1 (BLOC-1) and plays a role in intracellular vesicle trafficking, neurotransmitter release [28]. Male germ cell-associated kinase (MAK) is a serine/threonine protein kinase involved in the cell cycle. It expresses primarily in germ cells. However, the function of DTNBP1 and MAK involved in liver cells of HDL deficiency needs to be studied further.

### Analysis of the data of Mouse Embryonic Stem Cells [20]

Zhou et al. [20] conducted the whole genome expression to identify the gene network in mouse embryonic stem cells (ESC). Two major properties of ESC are pluripotency and capability of propagating indefinitely [29]. ESC cells form the inner embryo mass of blastocyst by proliferation and have the potential to differentiate into specific cell type for the development of embryo under an extremely strict control [30]. The regulation of transcription factors and the interaction with epigenetic factors form a gene regulatory network to control the pluripotency and differentiation of ESC cells. Four transcription factors (TFs), Oct4, Sox2, Klf4 and c-Myc, play an important role of maintaining ESC cells at the pluripotent state [31]. Additional TFs are also involved in the regulation of ESC [32]. An ESC cell is also capable of proliferating and self-renewal [29]. Thus, ESC cells are widely used in developmental biology and stem cell research [33].

In Zhou et al. [20], the status of mouse ESC was assessed by sorting the expression level of Oct4-GFP mouse ESC (mESC) using flow cytometry. With a non-GFP mESC line as a negative control, they studied the status of mESC by the expression level of the Oct4 gene. The total RNA samples were extracted from 16 sorted cell samples to study the expression profiles on Genome 430 V.2 Affymetrix microarrays. Eight profiles were marked as Oct4 positive (Oct4+), implying that the cells retained the attributes of ESC. Other 8 profiles were sorted with low Oct4 expression (Oct4-). The analysis was performed by dChip, a microarray analysis tool developed by Li and Wong [34]. The fold change was calculated by the expression level in Oct4+ samples over that in Oct4- samples, and noted as the ratio R. The *p*-value of the average difference between two groups was calculated by the Welch modified two-sample *t*-test. The Oct4-sorted + subset was defined as the ones with R > 2 and p-value <0.05, while the Oct4-sorted- subset as the ones with R <0.5 and *p*-value <0.05.

With the DAR algorithm, we first transformed and discretized the data to 1, 0, and −1, with the same criterion (R >2 or R < 0.5) as in Zhou et al. [20]. The average probe hybridization intensity of the 8 Oct4- samples served as the baseline for each gene. Next, every data point was divided by the baseline in that probe. If the ratio R was larger than 2, the data point was labeled as ‘1’; if it was smaller than 0.5, it was labeled as ‘-1’; all other data points were labeled as ‘0’. All gene symbols and RefSeq annotation were obtained from the supplement data of Zhou et al. [20]. The aim of this analysis was to figure out the significantly different genes in expression level between Oct4+ and Oct4-. Since the normalization is based on the average expression level of Oct4- for discretization, the significant association rule in ‘1 to Oct4+’ is sufficient to signify that the expression level of Oct4+ is significantly larger than that of Oct4-, and it can be considered as the genes in the Oct4-sorted + subset in Zhou et al. [20]. Similarly the significant rule ‘-1 to Oct4+’ means that the expression level of Oct4+ is significantly lower than that of Oct4-.

**Fig. 4:**
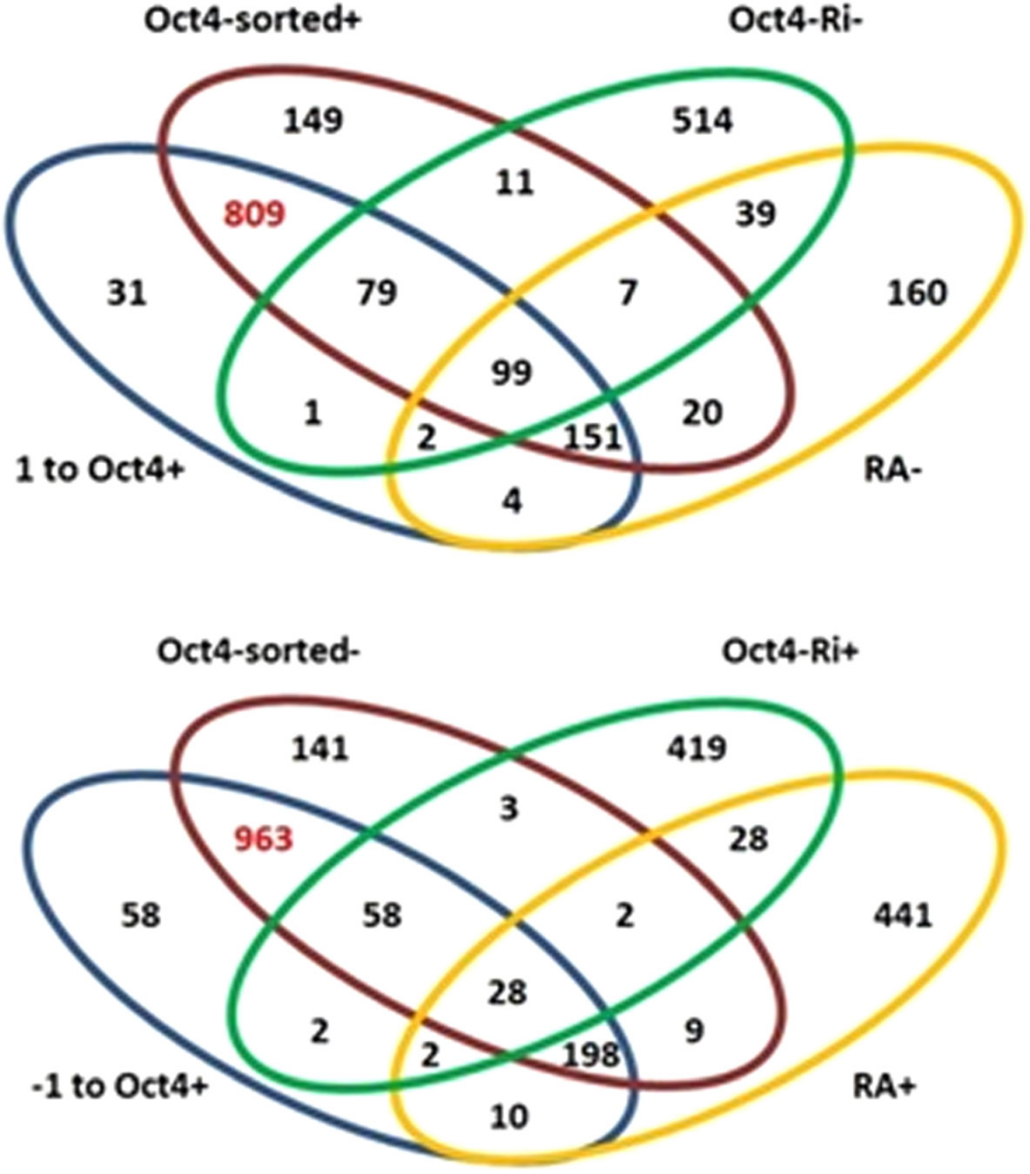
Overlaps between the gene sets in significant association rules, Oct4-sorted, Oct4-RNAi, and RA-induction. The number in the Venn diagrams shows the intersection set of the contiguous regions

For the mouse ESC dataset, there were 2037 significant association rules in ‘1 to Oct4+’, while the subset ‘-1 to Oct4+’ had 2491 significant rules under the 95 % confidence interval of support and confidence. In Fig. 4, there were four subsets for each Venn diagram, respectively. The lists of differentially expressed genes from expression profiles that were treated by Oct4 RNAi knockdown and retinoic acid (RA) induction were reported from Ivanova et al. [31]. The expression of Oct4 RNAi suppresses the level of Oct4 in ESC, so Oct4 Ri + can be considered as similar to Oct4- in Zhou et al. [20]. In comparison, the expression level of Oct4 in ESC remains relatively high without the expression of Oct4 RNAi, which is comparable to the samples of Oct4+ in Zhou et al. [20]. Expression of retinoic acid (RA+) induces differentiation of ESC into specific cell types. The gene regulation of RA+ can also be compared to the samples of Oct4- in ESC [35]. After excluding the redundancy by RefSeq ID and eliminating the blanks, there were 1176 significant genes in the subset of ‘1 to Oct4+’. Compared to the 1325 genes listed in the Oct4-sorted + subset, there were 1138 genes (96.77 % of the ‘1 to Oct4+’ gene subset) that appeared in both subsets. Out of the 1138 intersected genes, 809 (71.09 %) genes are shared with the Oct4-sorted + subset. On the other hand, subset Oct4-sorted- and subset ‘-1 to Oct4+’ contain 1440 and 1319 genes, respectively, and the two subsets shared 1247 genes, which contain 94.54 % of the ‘-1 to Oct4+’ gene set. There were 963 out of the 1247 (77.22 %) genes shared between Oct4-sorted- and ‘-1 to Oct4+’. The high proportion of intersection indicates that the association rules select significant genes efficiently and have good reproducibility to the subsets listed by t-test. JARID2 was listed in the unique genes (including 31genes) that were identified only by the association rule approach. JARID2 is an AT rich interactive domain-containing protein that functions as a DNA-binding protein [36]. Pasini et al. [37] demonstrated that JARID2 could recruit the Polycomb repressive complex 2 (PRC2), which plays a crucial role in regulating gene expression essential for development and differentiation in pluripotential cells [38], to facilitate histone methylation. The additional genes that the DAR algorithm identified in this data set need further experimental analyses to address the roles of genes that involved in the regulation of ESCs.

Zhou et al. [20] chose 7 genes as ESC and 7 differentiated cell markers. Oct4, Sox2, Nanog, Esrrb, Tcl1, Dppa5, and Utf1 showed more than 9 fold changes in the positive direction in the raw data and were selected as ESC markers in the significant list of Oct4-sorted+. We also apply multiple *t* test with adjusted p-value. The results are listed in Table 5. As shown in Table 5, six out of the seven chosen genes were significant in the subset of ‘1 to Oct4+’. The confidence level of Sox2 failed to pass the minimum support and minimum confidence under the 95 % confidence interval. For the differentiation markers, all seven genes were listed in the subset of “Oct4-sorted-” and expressed higher than 10 fold changes in the negative direction. However, the test of Tgfbr3 (0.5 in term of support) does not pass the minimum of support (0.5031) in association rule ‘−1 to Oct4+’ under the 95 % confidence interval because high variation of expression level among the eight Oct4- samples gave rise to a low support level in the ‘−1 to Oct4+’ rule. In terms of confidence, Tgfbr3 (0.8) did pass the minimum confidence (0.7735) at the 95 % confidence level. If we adjusted the criterion to the 90 % confidence level, reducing the minimum support to 0.461 and the minimum confidence to 0.707, Tgfbr3 would be significant. This indicates that the criteria should be chosen properly depending on the type and the property of the dataset. Moreover, Zhou et al. [20] listed 15 regulators that were involved in the states of ESC (Table 6). Eleven of the 15 genes are significant in both gene subsets. Out of the four insignificant genes, Stat3 and Sall4, that were not significant in all association rules, were also excluded from significant subsets in the original paper. Otx2 was as insignificant as Sox2 in the rule of ‘1 to Oct4+’ with the 95 % confidence level.

**Table 5:** The results of the marker genes of ESC or differentiation from the dataset of Zhou et al. [20]

Marker genes of ESC	RefSeq ID	Sig. in Oct4-sorted+	Sig. in’1 to Oct4 + ’	Multiple *t*-test (adj. *p*-value)
Oct4	NM_013633	+	+	4.00E−04
Sox2	NM_011443	+	-	6.00E−04
Nanog	NM_024865	+	+	0.0038
Esrrb	NM_011934	+	+	6.00E−04
Tcl1	NM_009337	+	+	4.00E−04
Dppa5	NM_025274	+	+	4.00E−04
Utf1	NM_009482	+	+	4.00E−04
Marker genes of differentiation		Sig. in Oct4-sorted-	Sig. in ‘-1 to Oct4 + ’	
Tcf7l2	NM_009333	+	+	0.1526
Gata4	NM_008092	+	+	6.00E−04
Gata6	NM_010258	+	+	0.0032
Tgfbr3	NM_011578	+	-	1
Foxa2	NM_010446	+	+	4.00E−04
Bmp2	NM_007553	+	+	0.017
Cited2	NM_010828	+	+	4.00E-04

**Table 6:** The results of the identified core regulators from the dataset of Zhou et al. study [20]

Gene name	RefSeq ID	Sig. in Oct4+ sorted	Sig. in ‘1 to Oct4+’
Oct4	NM_013633	+	+
Sox2	NM_011443	+	-
Nanog	NM_024865	+	+
Stat3	NM_213659	-	-
Esrrb	NM_011934	+	+
Sall4	NM_201395	-	-
Nr5a2	NM_030676	+	+
Otx2	NM_144841	+	-
Tcf7	NM_009331	+	+
Etv5	NM_023794	+	+
Utf1	NM_009482	+	+
Tcfap2c	NM_009335	+	+
Mtf2	NM_013827	+	+
Rest	NM_011263	+	+
Rbpsuh	NM_009035	+	+

### Analysis of the Expression Profiles among the Cohort of Leukemia Patients [21]

The classification of tumor types has been a challenge for pathologists to decide a specific cancer treatment for patients. The knowledge-based molecular markers are critical for the clinical treatment. Acute leukemia has been classified into acute lymphoblastic leukemia (ALL) and acute myeloid leukemia (AML) by enzyme-based histochemical diagnosis [39, 40]. In 1999, Golub et al. introduced gene expression profiling as an approach to validate the outcome of clinical diagnosis [21]. Thirty-eight acute leukemia cases from diagnosed patients (27 ALL, 11AML) were acquired in that study. Thirty-eight total RNA samples extracted from acute leukemia patients’ bone marrow were hybridized to Affymetrix microarrays with probes for 6817 human genes.

Identifying differentially expressed genes with statistical significance has been a crucial step of microarray analysis. Since a large number of replicates in one treatment group of acute leukemia was conducted [21], the gene expression data set has been used as a good training set for statistical method development. Many statistical methods have been published for identifying differential gene expression in microarray [41]. Here we compare the DAR algorithm to one of the methods listed in Kim et al.’s paper. The lists of significant genes were obtained by the *t*-test and by our proposed association rule approach.

The normalization of the raw data was conducted for each sample by subtracting its median and dividing it by its quartile range. The *t*-statistic is provided in Pan [42], which is used to identify differently expressed genes between two groups with a 95 % confidence level. For the association rule method, after normalization, the discretization step follows the Transitional State Discrimination method (TSD) in Pati and Das [43]. For each gene, we subtracted its mean and divided it by its standard deviation. If the normalized value was ≧1, we labeled it as 1; if it was ≦ − 1, we labeled it as −1; otherwise, we label it as 0. Based on these labeled data, the proposed procedure can be applied to find out the significant rules with a 95 % confidence level.

In order to mine the biological information in the final gene list, pathway analysis was conducted to map these genes to known pathways (using Gene Ontology terms as proxy). If the genes set is too small, some potential significant pathways may not be discovered. Therefore, unlike Pan [42], we did not apply the *p*-value adjustment in the multiple comparisons when selecting significant genes.

With the 95 % confidence level, there were 683 significant association rules in ‘ + 1 to AML’ and 467 in ‘−1 to AML’. Because no significant rules in ‘ + 1 to ALL’, ‘−1 to ALL’, ‘0 to AML’ and ‘0 to ALL’ were found at the 95 % confidence level, the rule of’ + 1 to AML’ can be interpreted as AML > ALL, whereas the rule of’−1 to AML’ as ALL > AML. The significant gene lists selected by *t*-test with α = .05 had 97 probes in AML > ALL and 672 probes in ALL > AML (Table 7). For the significant gene in AML > ALL, our association rule had 41 out of the 86 non-redundant genes in common with the gene list selected from t-test. As for ALL > AML, 201 out of 615 non-redundant genes were reported by both statistical methods. The fixed number of degree of freedom in t-test could be the factor to the agreement of acquiring genes between association rule and t-test. Table 8 listed over-represented pathways in Gene Ontology (GO). Although a low proportion of common genes were reported by both methods, the gene list from association rule in ALL > AML and those from t-test have a strong agreement in pathway analysis. Among 19 significant GO pathways (*p*-value <0.05) in the over-representation test by using the significant gene list from the association rule, 16 pathways were also reported as over-representation pathways from the t-test significant gene list (Table 8). This indicates that the pool of genes in both methods play similar roles in the biological functions even though the number of common genes was low. That is, most of the genes in these two subsets, even the common set is small, involve in similar pathways in the cells. The significant pathways in both gene lists of AML > ALL did not have many overlaps simply due to the limited gene list selected by the t-test, giving rise to only one significant pathway found in pathway analysis. Furthermore, the significant biological processes, e.g., hemopoiesis and developmental process indicate the fundamental differences of cell regulation between myeloid and lymphoid cells according to the overrepresentation test of gene list that was identified by the association rule approach. Nevertheless, it is worth noticing that this comparison had relatively minor input to the biology and clinic since there is lack of a cohort of baseline to calibrate the expression profile between AML and ALL samples.

**Table 7:** Number of significant probes and non-redundant genes under tested methods

	Association rule	*t*-test	Common gene
^aAML >ALL (# of probe)^	576 (683)	86 (97)	41
^bALL >AML (# of probe)^	396 (467)	615 (672)	201

^a^Number of Ensembl genes that was expressed higher significantly in AML than in ALL (non-redundant)

^b^Number of Ensembl genes that was significantly up-regulated gene in ALL than in AML (non-redundant)

**Table 8:** Significant pathway in term of biological process of GO database

ALL > AML (−1 to AML) in association rule		ALL > AML in *t*-test	
Biological Process	*P*-value	Biological Process	*P*-value
**Metabolic process**	5.05E–25	**Metabolic process**	8.38E–25
**Primary metabolic process**	1.65E–21	**Primary metabolic process**	2.80E–23
**Nucleobase-containing compound metabolic process**	2.20E–13	**Nucleobase-containing compound metabolic process**	3.20E-21
**Cell cycle**	5.27E–07	**DNA metabolic process**	3.23E–16
**Protein metabolic process**	1.30E–06	**Cell cycle**	1.41E–13
**DNA metabolic process**	3.88E–06	**DNA repair**	7.06E–10
**Cellular component organization or biogenesis**	1.59E–04	**DNA recombination**	6.09E–08
**Cellular process**	2.00E–04	**Cellular process**	1.10E–07
**Chromatin organization**	7.37E–04	**DNA replication**	4.00E–07
**RNA metabolic process**	1.01E–03	Response to stimulus	4.04E–07
**Organelle organization**	3.10E–03	mRNA processing	2.10E–06
**Cellular component organization**	5.49E–03	**RNA metabolic process**	2.70E–06
**DNA repair**	7.40E–03	**Cellular component organization or biogenesis**	5.52E–06
Protein complex biogenesis	1.61E–02	response to stress	6.65E–05
Protein complex assembly	1.61E–02	**Cellular component organization**	1.73E–04
**DNA recombination**	3.09E–02	**Protein metabolic process**	2.75E–04
**DNA replication**	3.10E–02	**Organelle organization**	3.43E–04
Transcription from RNA polymerase II promoter	4.21E–02	Purine nucleobase metabolic process	8.90E–04
**Transcription, DNA-dependent**	4.84E–02	Protein phosphorylation	1.03E–03
		mRNA splicing, via spliceosome	1.40E–03
		Meiosis	3.33E–03
		**Chromatin organization**	4.12E–03
		RNA splicing	8.94E–03
		RNA splicing, via transesterification reactions	8.94E–03
		Cellular defense response	2.28E–02
		Cell proliferation	3.93E–02
		Nitrogen compound metabolic process	4.54E–02
		**Transcription, DNA-dependent**	4.63E–02
		Regulation of carbohydrate metabolic process	4.66E–02
AML > ALL (+1 to AML) in association rule		AML > ALL in *t*-test	
Biological Process	*P*-value	Biological Process	*P*-value
Metabolic process	1.20E-10	**Cellular process**	1.14E-02
Cell communication	8.26E-10		
Developmental process	3.19E-09		
**Cellular process**	5.32E-09		
Immune response	6.40E-09		
Immune system process	4.00E-08		
Primary metabolic process	5.37E-08		
Macrophage activation	1.43E-07		
Response to stimulus	1.71E-07		
System development	8.39E-06		
Cell death	6.55E–05		
Apoptotic process	6.55E–05		
Death	7.05E–05		
Proteolysis	2.85E–04		
Hemopoiesis	6.24E–04		
Protein metabolic process	1.30E–03		
Negative regulation of apoptotic process	1.51E–03		
Transport	4.90E–03		
Localization	8.73E–03		
Biological regulation	9.95E–03		
Angiogenesis	1.46E–02		
Regulation of biological process	2.34E–02		
B cell mediated immunity	7.41E–02		
Skeletal system development	8.16E–02		
Cellular defense response	8.65E–02		
Mesoderm development	9.51E–02		

Bolded terms indicate the significant pathways appeared in both DAR and *t*-test

### An Application to the RNA-seq Data of Genomic Imprinting Study [22]

Genomic imprinting is an epigenetic system that is inheritable from parents in diploid organisms. It is important for mammalian development and embryonic growth. The epigenetic tags on imprinted genes of a one-cell embryo were fully established during the paternal and maternal germ cell developmental process [44,45]. Dynamic reprogramming, such as active and passive demethylation of both parental genomes, is crucial for imprint maintenance throughout development [46–48]. During the formation of gonads in the embryo, primordial germ cells (PGCs) undergo epigenetic erasure to recover pluripotency [49]. According to previous studies, gene Tet1 may be involved in the erasure of genomic imprinting in the PGCs in E11.5- E13.5 mouse embryos [50, 51]. Yamaguchi et al. [51] found that the paternal^KO^ (Tet1^−/−^ male x wild-type female) mice had significantly fetal and postnatal growth defects. To link the phenotypic changes to the dysregulated erasure of imprinted genes on paternal alleles, Yamaguchi et al. [22] performed the RNA-seq analysis of E9.5 embryonic PGCs on 10 Tet1 paternal^KO^ and three control mice. The normalized expression level was calculated in terms of FPKM and was log2 transformed. The cutoff of significantly altered genes of the paternal^KO^ compared with the average expression level of the control samples was the fold-change of 1.5. We followed the criteria of Yamaguchi, et al. [22] to discretize the data. If the expression level of the gene was more than 1.5 times over the average expression level of control samples, it was labeled as 1, whereas if the expression level was less than two-third of the average expression level of the control samples, it was labeled as −1. Otherwise, it was labelled as 0.

The 75 % confidence level was set to select genes in the DAR algorithm. There were 575 meaningful rules found in the rule of “1 geneA implies paternal^KO^”, representing the significantly up-regulated genes in paternal^KO^ mice. Four hundred and fifty-six meaningful rules were found in “-1 geneA implies paternal^KO^”, which represent significantly down-regulated genes (Table 9). In Yamaguchi et al. [22], 905 up-regulated and 635 down-regulated genes were identified. The percentages of overlaps in up-regulated and down-regulated genes between the two studies are 27.48 % and 28.29 %, respectively. In Yamaguchi et al. [22], genes were identified as significant as long as at least two of the ten paternal^KO^ embryos analyzed had a fold-change (FC) larger than 1.5 times to the average of the control embryos. The stringency of selecting rule from the original paper was relatively relaxed than it was in our method. This may be the reason why the overlaps of the resulting genes from two methods are small.

**Table 9:** Number of significant DAR rules using five different confidence levels

Association rules	New method				
	70 %	75 %	80 %	85 %	90 %
	Confidence	Confidence	Confidence	Confidence	Confidence
Expression of rules	Number of rule (number of overlapping genes in Yamaguchi et al.’s study)				
1 geneA - > case	1055 (267)	575 (158)	168 (38)	34 (6)	9 (0)
−1 geneA - > case	869 (255)	456 (129)	117 (30)	11 (2)	5 (1)
1 geneA - > control	609 (9)	581 (9)	304 (5)	304 (5)	282 (5)
−1 geneA - > control	737 (8)	732 (8)	332 (3)	332 (3)	320 (3)
0 geneA - > case	236 (2)	125 (2)	36 (36)	2 (0)	0
0 geneA - > control	2478 (701)	2370 (696)	1474 (444)	876 (238)	454 (132)

Another list of expressed imprinted genes was ranked by the FPKM value. In Yamaguchi et al. [22], 81 expressed genes were found to be related to the regulation of genomic imprinting. By applying the same criteria of being a significant gene (at least two out of ten have FC > 1.5), we found 39 genes to be significantly expressed in paternal^KO^ mice (Table 10). Applying our proposed method, we found 8 out of the 39 genes to be marked as significant association rules either in “1 geneA implies paternal^KO^” or “−1 geneA implies paternal^KO^”. The number of genes shared by the two methods is low. The criteria to select the genes in Yamaguchi et al.’s study [22] did not have statistical support and only considered if two individuals had a FC larger than 1.5. The loose criterion may explain the low overlaps. Furthermore, some of the genomic imprinting related genes were assigned as activated and inhibited significantly at the same time (e.g., Peg3, Airn, Begain, Sfmbt2 in Table 10) based on the criteria of the paper of Yamaguchi et al. [22]. Such genes were considered as ambiguous if one only mines significant single antecedent rules. However, in theory they can be captured if we consider higher leveled antecedent rules mining when the genes have significant interaction with the other significant genes. The multiple *t* test with adjusted *p*-value has been applied to analyze as well. The results has been listed in Table 10.

**Table 10:** Expressed imprinted genes in the RNA-seq data of Yamaguchi et al. [22]

Gene symbol	Expressing allele	at least two out of ten FC > 1.5		
		Significant (up, down) or not	Intersect with significant association rules	Multiple t-test (adj. p-value)
Mest	P	Down		1
H19	M	Up		1
Meg3	M	Down		1
Grb10	M	Up	1 geneA - > paternal^KO^	1
Rian	M	Down		1
Peg10	P	Down	−1 geneA - > paternal^KO^	1
Cdkn1c	M	Up		1
Peg3	P	up/down		1
Sgce	P	Down	−1 geneA - > paternal^KO^	1
Asb4	M	Up	1 geneA - > paternal^KO^	1
Cmah	M	Down		1
Impact	P	Down		1
Pon2	M	Down		1
Ube3a	M	Up		1
Peg13	P	Down		1
Phlda2	M	Down		1
Dcn	M	Up		1
Airn	P	up/down		1
Ddc	P	Down		1
Zim1	M	Up		1
Magel2	P	Down		1
Begain	P	up/down		1
Tspan32	M	Down	−1 geneA - > paternal^KO^	1
Art5	M	Down		1
Wt1	M	Up		0.818182
Qpct	M	Down		1
Atp10a	M	Down		1
Nespas	P	Down	−1 geneA - > paternal^KO^	1
Tnfrsf23	M	Down		1
Tfpi2	M	Down		1
Sfmbt2	P	up/down		1
Nap1l5	P	Up	1 geneA - > paternal^KO^	1
Slc22a18	M	Down		1
Th	M	Up	1 geneA - > paternal^KO^	1
Usp29	P	Down		1
Cntn3	M	Down		1
Mst1r	M	Down		1
Calcr	M	Down		1
Kcnq1	M	Down		1

### Microarray Quality Control (MAQC) data sets [52]

In 2006, MicroArray Quality Control (MAQC) consortium launched a series of analyses across different platforms of microarrays [52]. They used four defined samples to acquire the expression profiles from eight commercialized microarray platforms. The overall objective of this project is to evaluate the sensitivity and reproducibility among microarray platforms. Those samples include 100 % Universal Human Reference RNA (UHRR, sample A), 100 % Human Brain Reference RNA (HBRR, sample B), 75 % UHRR: 25 % HBRR (sample C) and 25 % UHRR: 75 % HBRR (sample D). Five replicates of every sample were tested on one chip in one platform and three chips of every platform were conducted in this project. This resulted in 15 replicates for every sample in one microarray platform. In the same project, they also used three qPCR assays to validate 1297 genes selected from the common probes among microarray platforms. According to the single gene quantification study [53], the expression level qPCR can serve as great standard to evaluate the sensitivity and accuracy of microarray platforms. In total, they quantified 997 genes with TaqMan Gene Expression Assays (TAQ), 205 genes with Standardized RT (Sta) RT-PCR assays (GEX) and 244 genes with QuantiGene assays (QGN). The number of genes is 1297 in union [53].

In order to compare different statistical methods for determining differentially expressed genes (DEGs), Kadota and Shimizu [54] selected TAQ and GEX assay data to compare the DEG determined by *t*-test and Average Difference (AD, as known as log-fold-change) methods. They further used Benjamini and Hochberg approach to calculate the false discovery rate (FDR) for the p-value from the *t*-test [55]. They selected five microarray platforms from MAQC project [52] including Applied Biosystems (ABI), Affymetrix (AFX), Agilent Technologies for one-color array (AG1), GE Healthcare (GEH) and Illumina (ILM). Among these platforms, they compared the specificity and sensitivity of gene ranking methods for analyzing two-class data: weighted average difference (WAD), average difference (AD), fold change (FC), rank product (RP), moderated t statistic (modT), significance analysis of microarrays (samT), shrinkage t statistic (shrT), and intensitybased moderated t statistic (ibmT) [56].

**Fig. 5:**
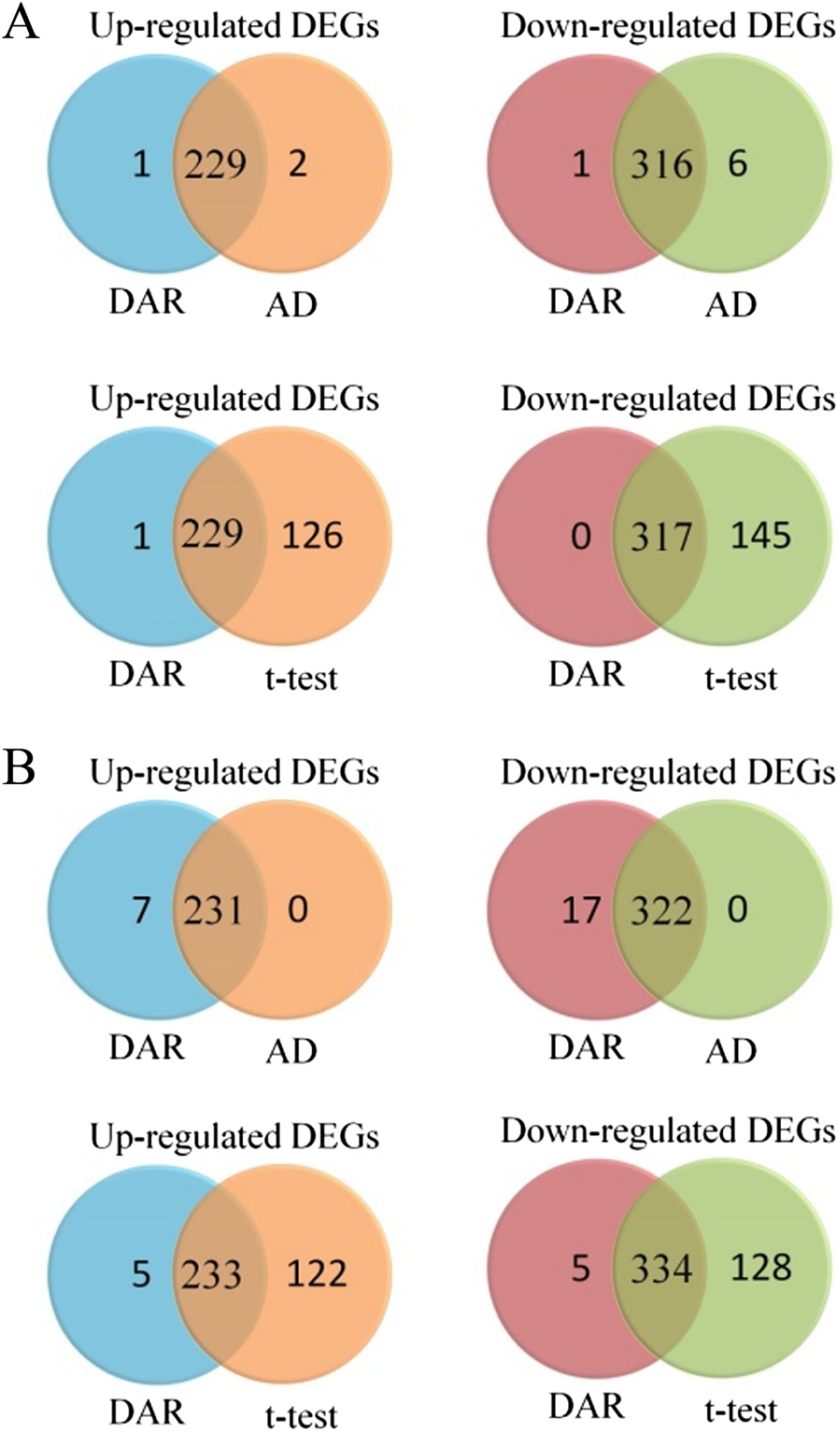
Number of common genes between DAR and compared methods in TAQ data. **a** DAR method with 90 % confidence level; **b** DAR method with 70 % confidence level

We tested the reproducibility of DEGs by dynamic association rule (DAR) with TAQ data, and selected ILM microarray assay to screen the DEGs by DAR and compared the result with three gene ranking methods in Kadota and Shimizu’s work [54]. The TAQ data had four replicates for sample A and four for sample B. In that study, the average expression level of sample B was divided by that of sample A to calculate the significant DEGs if the absolute value of AD was larger than one. The DEGs were selected in *t*-test when the FDR is smaller than 0.05. To select the significant DARs from TAQ data, we discretize the TAQ data by dividing gene expression level of each replicate by the average expression level of sample A that is similar to the calculation of AD method. The genes in TAQ data were assigned as up-regulated gene if the average expression level of sample B is larger than that of sample A, and labeled as down-regulated gene if the average expression level of sample A is larger than that of sample B. 70 % and 90 % confidence level were used to carry out the lists of DEG by DAR method. The result shows that almost all genes that listed as DEG by DAR method with 90 % confidence level were included in the lists of DEG by AD and *t*-test method (Fig. 5). The lists of DEG by DAR method with 70 % confidence level covered all genes listed in AD method and had several unique genes against the lists of DEG by *t*-test method in up-regulated DEGs and down-regulated DEGs.

The normalized ILM microarray data set that had 47,293 detecting probes was obtained from the published work of Kadota and Shimizu [54]. It left 23,080 probes after excluding non-detectable probes, which determined by all 30 detections having flag detection value less than 0.99. The WAD and FC statistic were re-calculated following the description in Kadota et. al’s study [56]. In brief, the FC for each gene was calculated as average non-log expression value for 15 replicates of sample B divided by the average expression value for 15 replicates of sample A. The WAD statistic was calculated as average log value of sample B divided by that of sample A, and weighted by the range of average log value for 30 detections value across all ILM detectable probes [56]. The discretization for DAR method was calculated as the log expression value of each replicate divided by the average log expression value of sample A. The cutoff of absolute value larger than 1 was used for categorization. Among 23,080 probes, 2775 DARs were found to pass the minimum support and minimum confidence in rule’1 geneA implies to sample B’ under 99 % confidence level, which could also be inferred as up-regulated genes. There were 3339 DARs were found in rule’-1 geneA implies to sample B’, and interpreted as down-regulated genes.

Since the WAD and FC statistic were used only for ranking, it did not assign a cutoff for DEG in Kadota et al.’s work [54]. Here we simply selected top 2775 probes and bottom 3339 probes for comparison after ranked with WAD and FC statistic. The intersection number of up-regulated and down-regulated probes between DAR and FC was 2668 (96.14 %) and 3139 (94.01 %). The intersection number of up-regulated and down-regulated probes between DAR and WAD was 2173 (78.31 %) and 2596 (77.75 %).

## Conclusions

Our association rules can be applied to gene expression data analysis with high confidence and reproducibility. We took advantage of the well-established association rule mining technique from marketing to develop an improved method and algorithm, the DAR algorithm, to mine significant association rules between gene regulation and trait. In particular, we derived the distributions of support and confidence for the association rule “if A then B” under the assumption of independence between A and B. Based on these distributions, we could then determine the minimal support and the minimal confidence. That is, for any association rule to be meaningful, their minimal support and minimal confidence must be higher than the theoretical upper limits under the independency assumption. While finding important association rules remains a challenging problem, we provided a formal procedure for testing whether a rule is meaningful. Certainly, when other sources of information is possible (from knowledge domain, for example), it is possible that an association rule is meaningful, even without beating this minimal value. Our conclusion here is purely data driven.

The issue of multiple hypothesis testing has been ignored here. The theory developed here would generally be applicable to test any given value on “Is this value meaningful?” In many applications, a very large number of association rules are searched. The following minimal support and confidence may be called for. Given a pre-specified value of minimal support s for finding all association rules “if A then B”, we can back solve the equation for screening purpose - this can save a significant amount of time. For example, from the Method section, given s and A (thus n and p_1_), we have

$$ {\mathrm{p}}_1\cdot {\mathrm{p}}_2+{\mathrm{z}}_{\mathrm{a}}\sqrt{\frac{{\mathrm{p}}_1{\mathrm{p}}_2\left(1-{\mathrm{p}}_1{\mathrm{p}}_2\right)}{\mathrm{n}}} = \mathrm{s} $$

and thus

$$ \left(\mathrm{n}+{\mathrm{z}}_{\mathrm{a}}^2\right){\mathrm{p}}^2-\left(2\mathrm{ns}+{\mathrm{z}}_{\mathrm{a}}^2\right)\mathrm{p}+{\mathrm{ns}}^2=0 $$

where *p* = p_1_ · p_2_ .

Suppose p∗ is the root for the above equation, then p_2_ must be larger than p∗ / p_1_ for the rule to be meaningful. Thus, we can ignore all events B whose p_2_ is smaller than p∗ / p_1_.

The analysis results from the four datasets used above show the power of assessing gene regulation by the DAR algorithm. The influential genes relative to HDL deficiency are distinguished from EST-based microarrays. The regulatory network of ESC including a few crucial transcription factors is also revealed by well-defined mouse microarrays. The agreement of the pathway analysis is evident between the association rule algorithm and the *t*-test in the leukemia dataset, although the proportion of intersected genes is small between the two lists of significant genes. The association rule approach that applies to the RNA-seq data also identifies a set of significant gene lists that has a moderate agreement with the results from the source study [22]. In this analysis, we extended the established data mining technique of association rules borrowed from market basket analysis to transcriptome analysis. A method was developed to normalize, transform, discretize, and add identification techniques to the data. A computer program produces many association rules. Having the originally produced rules that numbered in the hundreds, the theoretical minimum support and theoretical minimum confidence were used to trim rules. The resulting rules were found to be statistically significant and left to the investigator to determine whether they are significant biologically. Traditional statistical techniques were employed to validate the finding of the association rules with mixed results mostly stemming from the unique makeup of the data. Association rules mining is an alternative approach to hypothesis development and can facilitate researchers to consider alternative genes and relationships that would otherwise be ignored in gene expression data analysis.

### Availability of supporting data

The code and dataset can be downloaded from www.mixturetree.net.

## Abbreviations

DAR: Dynamic association rule
NGS: Next generation sequencing
MAQC: Microarray quality control
RPKM: Reads Per Kilobase per Million mapped reads
FPKM: Fragments Per Kilobase per Million mapped fragments
HDL: High-density lipoprotein
ESC: Embryonic stem cells
Apo A1: Apolipoprotein A1
ESTs: Expressed Sequence Tags
SC5D: Sterol C5 desaturase
SPATA5L1: Spermatogenesis associated 5-like 1
CASP6: Caspase 6
oxLDLs: Oxidized LDLs
DTNBP1: Dystrobrevin binding protein 1
DPC: Dystrophin-associated protein complex
BLOC-1: Lysosome-related organelles complex 1
MAK: Male germ cell-associated kinase
mESC: Mouse ESC
Oct4+: Oct4 positive
Oct4-: Low Oct4 expression
RA: Retinoic acid
ALL: Acute lymphoblastic leukemia
AML: Acute myeloid leukemia
TSD: Transitional State Discrimination
GO: Gene Ontology
PGCs: Primordial germ cells
FC: Fold-change
UHRR: Universal Human Reference RNA
HBRR: Human Brain Reference RNA
FDR: False discovery rate
ABI: Applied Biosystems
AFX: Affymetrix
AG1: Agilent Technologies for one-color array
GEH: GE Healthcare
ILM: Illumina
WAD: Weighted average difference
AD: Average difference
RP: Rank product
modT: Moderated t statistic
shrT: Shrinkage t statistic
ibmT: Intensitybased moderated t statistic
